# Commentary: FTO obesity variant circuitry and adipocyte browning in humans

**DOI:** 10.3389/fgene.2015.00318

**Published:** 2015-10-23

**Authors:** Samantha Laber, Roger D. Cox

**Affiliations:** ^1^Mammalian Genetics Unit, Medical Research Council HarwellOxfordshire, UK; ^2^Department of Physiology, Anatomy and Genetics, University of OxfordOxford, UK

**Keywords:** adipocyte browning, genome editing, GWAS (genome-wide association study), FTO locus, IRX5, rs1421085, IRX3

Genome-wide association studies (GWAS) identified >90 loci containing genetic variants, many in intronic regions, associated with human obesity. Understanding how these variants regulate gene expression has been challenging. Claussnitzer et al. present a strategy for deciphering non-coding complex trait genetic associations which greatly advances their functional analysis.

The strongest genetic association with risk to polygenic obesity are single-nucleotide variants in intron 1 and 2 of the *FTO* (fat mass and obesity associated) gene (Yang et al., 2012; Locke et al., 2015). However, of the 89 genetic variants in FTO intron 1 and 2, the causal variants, and their mechanistic underpinning have been elusive. A new report by Claussnitzer et al. identified a causal variant (rs1421085) and revealed its function in preadipocytes. Their study, published in the *New England Journal of Medicine*, provides evidence for the rs1421085 T to C single-nucleotide alteration to result in a cellular phenotype consistent with obesity in primary human adipocytes, including decreased mitochondrial energy generation and increased triglyceride accumulation (Claussnitzer et al., 2015).

GWAS have identified >90 loci and thousands of single-nucleotide-polymorphisms (SNPs) associated with body mass index (BMI). The majority of these SNPs are in non-coding regions of the genome and therefore do not directly alter protein sequence which makes their interpretation challenging (Zhang et al., 2014). In fact, intronic GWAS signals, which characteristically encompass a large number of variants in linkage disequilibria, have very rarely lead to the discovery of the disease-causing variants or the mechanism by which they affect disease susceptibility. Interestingly, many GWAS regions are enriched for histone modifications (The ENCODE Project Consortium, 2012), suggesting they may play a role in gene regulation. Due to the time and tissue-specificity and the developmental and epigenetic complexity of gene regulation, functional approaches require the study of relevant human tissues and cells as well as bioinformatics approaches (e.g., the search for phylogenetic conservation) that reliably assess the regulatory role of non-coding variants (Claussnitzer et al., 2014).

Using a range of bioinformatics and genetic approaches across diverse human tissues and cell types, Claussnitzer et al. unraveled the functional circuitry of the *FTO* locus. They present a model that deciphered: (1) the relevant tissue and cell type; (2) the regulated genes; (3) the causative variant; (4) the upstream regulator; (5) the cellular phenotype; and (6) the organismal phenotype (Figure 1). Specifically, their study shows that intron 1 of *FTO* harbors a 10 kb potent super-enhancer which is active in the mesenchymal and adipocyte lineages. This enhancer region contains the variant rs1421085 in a highly conserved motif comprising a binding site for ARID5B. ARID5B when binding to this motif acts as a transcriptional repressor. The *FTO* risk allele disrupts binding of ARID5B thereby giving access to a super-enhancer active in preadipocytes, resulting in the over-expression of two genes distal to *FTO*, namely *IRX3* and *IRX5*. Ultimately this leads to a cell-autonomous shift from mitochondrial energy production to lipid accumulation in risk allele carriers. Strikingly, genome editing of primary human preadipocyte samples toward the protective allele rescued the associated cellular phenotype, causing brown-like adaptation in the adipocyte as shown by up-regulation of thermogenesis. In their study, Claussnitzer et al. show that decreased expression of IRX3 and IRX5 in pre-adipocytes caused them to develop into brown-like adipocytes, with increased mitochondrial uncoupling which was maintained in mature adipocytes. Using the aP2-CRE for adipose-specific IRX3 inhibition, they show that at the organismal level, adipose specific IRX3 knockdown in mice resulted in 57% reduced fat mass ratio and high-fat-diet induced obesity resistance. This effect was significantly larger compared to the hypothalamus-specific IRX3 inhibition (Ins2-IRX3DN) which resulted in 19% reduced fat mass ratio. These compelling results suggest that the rs1421085 single-nucleotide change can alter metabolism that is responsible for the association between the first intron of *FTO* and obesity in humans. Further, this study clearly identifies adipocyte precursor cells as one of the primary effectors of *FTO* locus activity.

**Figure 1:**
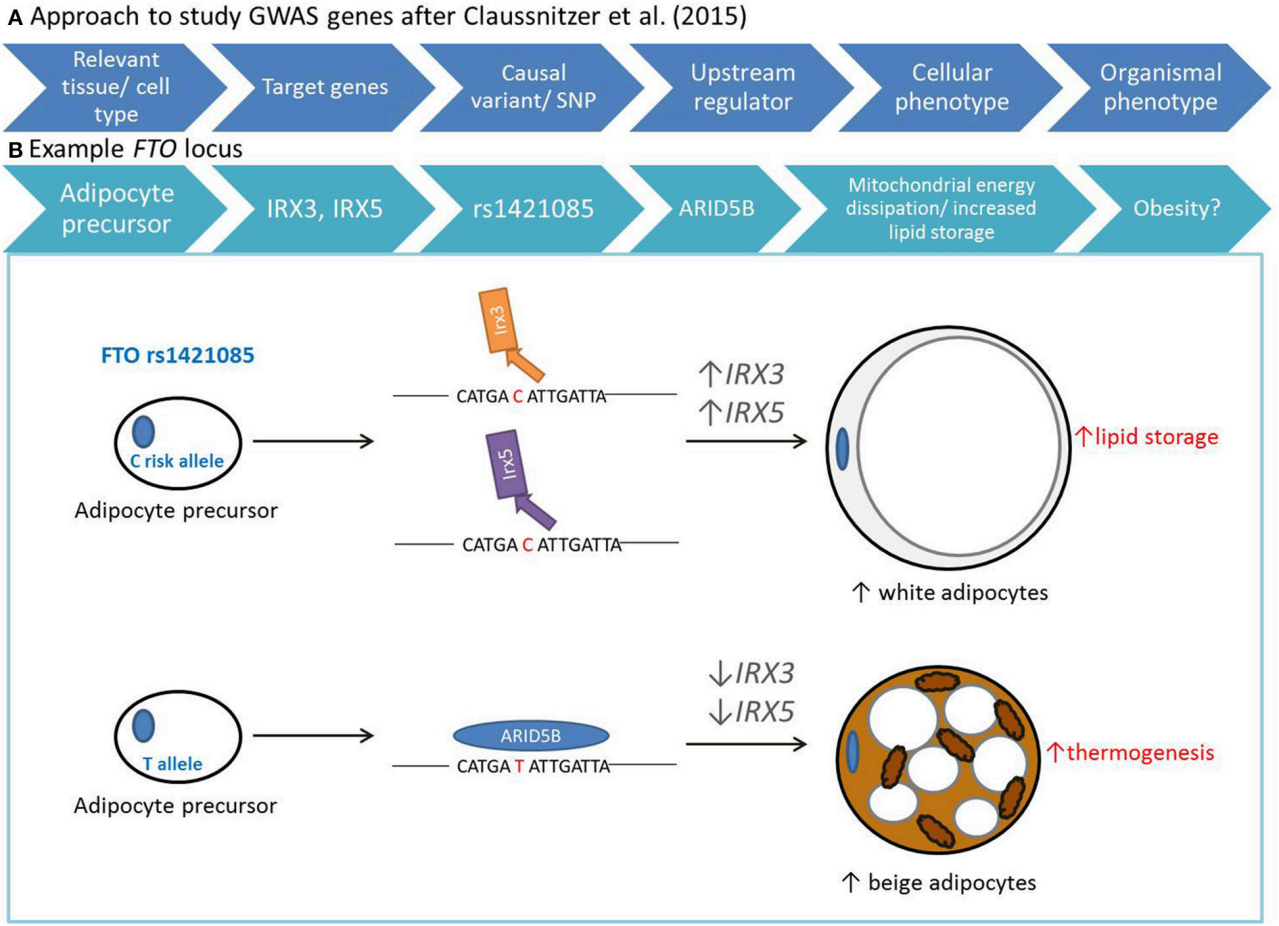
**Model for deciphering non-coding variants identified by GWAS after Claussnitzer et al. (2015)**. **(A)** Approach to study genes identified by GWAS involved establishment of relevant tissue and cell types, target genes, causative variants, upstream regulator, and cellular/organismal phenotypes. **(B)** With their work on the *FTO* locus, Claussnitzer et al. established that carriers of the *FTO* risk allele have a motif disruption in rs1421085 that leads to decreased binding of ARID5B, resulting in increased expression of IRX3 and IRX5 in pre-adipocytes. Increased expression of these genes shifts the development of these cells toward the “white program,” where mature cells have increased lipid storage. In contrast, the protective T allele results in adipocyte precursors to develop into beige adipocytes, characterized by increased thermogenesis.

Claussnitzer et al. identify the causal variant in the *FTO* locus and reveal its function specifically in preadipocytes, where the risk allele affects the basic anabolic function of the adipocyte, shifting it from substrate storage to fuel dissipation through increased mitochondrial uncoupling. What remains to be determined is the precise molecular mechanism by which IRX3 and IRX5 influence mitochondrial energy consumption and metabolism on an organismal level. Mouse models of increased IRX3 and IRX5 expression as well as *in vivo* models harboring the human mutations are necessary to fully understand the association between *FTO* intron 1 and obesity. A further intriguing question is whether additional conserved motifs and SNPs in *FTO*, other than rs1421085, can be functionally linked to disturbed metabolism and whether other tissues or cell types might be affected (Smemo et al., 2014). The FTO gene itself has recently been shown to play a role specifically in adipocyte precursors where it influences early processes in the differentiation cascade (Zhao et al., 2014; Merkestein et al., 2015) and FTO-knockout (in which a part of *Fto* exon 3 is deleted) results in a similar adipose phenotype as the IRX3-knockout, raising the question of the role of FTO itself in this cluster of adipocyte metabolism genes.

Demonstrated by their work on the *FTO* locus, Claussnitzer et al. present a strategy for deciphering non-coding complex trait genetic associations which can be used to functionally analyze GWAS hits in the future. In addition, this study very elegantly provides evidence for the clinical importance of mitochondrial activity and thermoregulation in white adipose tissue in obesity. By presenting a causative SNP, its regulator and a cellular phenotype, this study certainly provides new important insight and will guide future research into one of the most prominent obesity loci.

Claussnitzer et al. show that *FTO* obesity risk variants cell-autonomously and dynamically modulate mitochondrial activity of human white adipose tissue, a cellular phenotype consistent with obesity. This finding raises the possibility of thermogenesis in adipose tissue as a therapeutic target. Importantly, this study provides a convincing model for translating information arising from GWAS.

## Author contributions

SL and RC contributed to the discussion and writing of the manuscript.

## Funding

SL was supported by a MRC PhD studentship.

### Conflict of interest statement

The authors declare that the research was conducted in the absence of any commercial or financial relationships that could be construed as a potential conflict of interest.
